# Reconstruction of total upper eyelid defects using a myocutaneous advancement flap and a composite contralateral upper eyelid tarsus and hard palate grafts

**DOI:** 10.1016/j.jpra.2021.01.003

**Published:** 2021-02-05

**Authors:** Wendy W. Lee, Oded Ohana, Dimitra M. Portaliou, Benjamin P. Erickson, Mohamed S. Sayed, David T. Tse

**Affiliations:** a Department of Ophthalmology, Bascom Palmer Eye Institute, University of Miami, Miller School of Medicine, Miami, FL, United States; b Department of Ophthalmology, Guys and St Thomas’ NHS Foundation Trust, London, United Kingdom; c Department of Ophthalmology, Byers Eye Institute, Stanford university, Palo Alto, CA, United States; d Your Eye Specialists, Plantation, FL, United States

**Keywords:** Total upper eyelid defect, Hard palate, Posterior lamella, Reconstruction

*Dear Sir,*

Reconstruction of total upper eyelid defects pose a considerable challenge, requiring precise lamellar reconstruction to achieve proper eyelid function and aesthetics. Reconstruction techniques often require multiple surgeries, with blocking of the visual axis for varying periods of time before achieving the final result.^1^ Commonly used posterior lamella substitutes are contralateral tarsus and Hard-Palate-Graft (HPG). Contralateral tarsus availability may be limited by concurrent eyelid pathologies and previous surgery. A further requirement is to leave at least 4 mm of tarsal plate height to avoid destabilizing the eyelid. HPG thickness, lack of pliability and presence of keratinized epithelium can create a bulky eyelid appearance and might add to post-operative corneal complications.^2^ We describe a single sitting surgical technique for total eyelid reconstruction by using an upper eyelid preseptal myocutaneous advancement flap and a composite posterior lamella graft made of free contralateral tarsoconjunctival graft and HPG.

## Surgical technique

**Figure 1:**
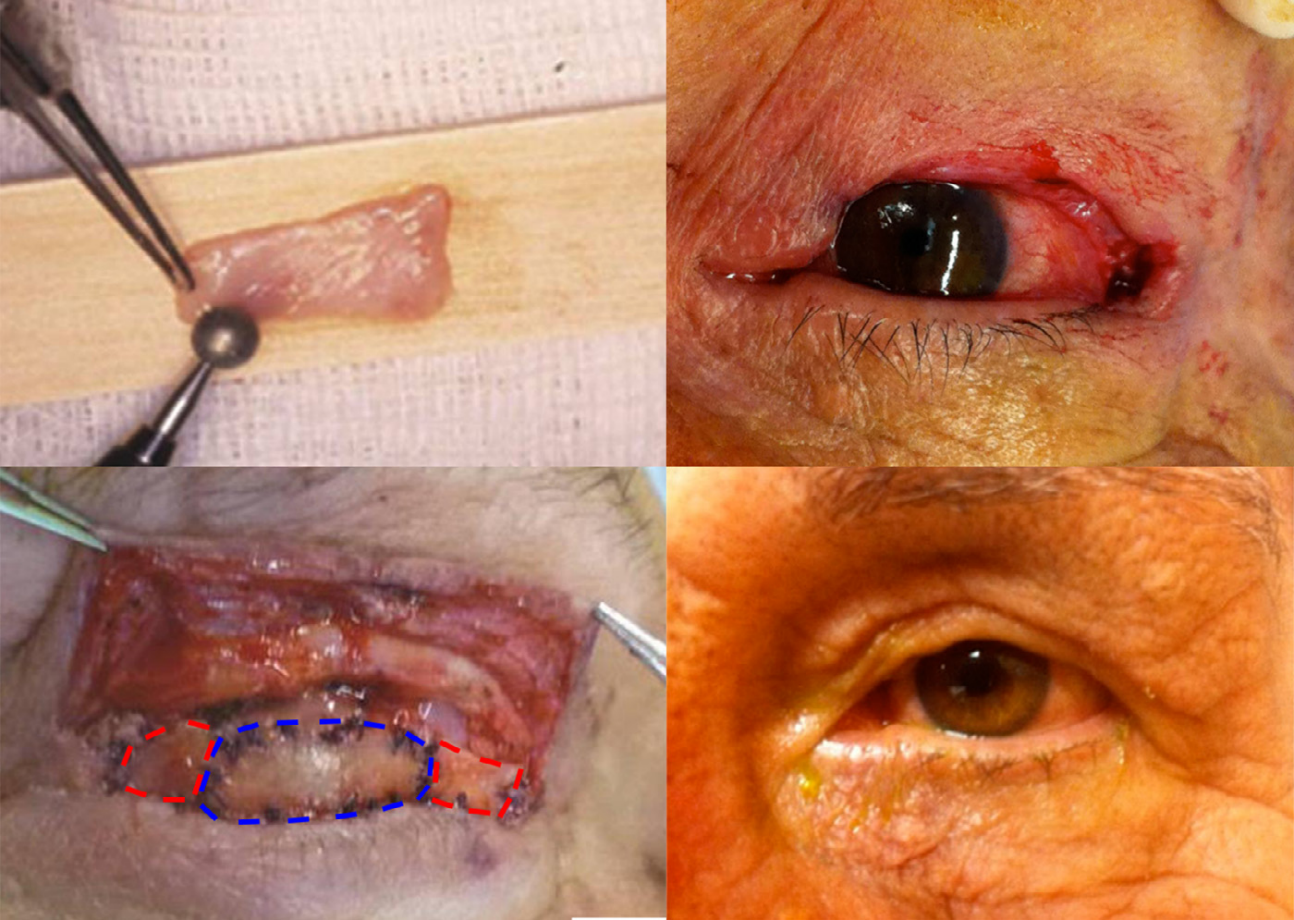
Composite Graft: *Top left: Hard palate graft*. A diamond burr is used to remove the keratinized mucosa of the hard palate graft and to thin it to 70% thickness. *Top right: Pre-Operative defect.* No residual upper eyelid tarsal plate is present, and the defect extends almost to the lateral orbital rim. *Bottom Left: Intra-Operative picture.* The composite graft sutured in place. A central contralateral free tarso-conjunctival graft is outlined in blue, while flanking hard palate grafts are outlined in red. *Bottom right: Postoperative appearance.* No ptosis or eyelid bulkiness.

First, we address the posterior lamellar defect: A full-thickness tarso-conjunctival graft is harvested from the contralateral upper eyelid, leaving at least 4 mm of tarsal plate height to minimize donor site morbidity. This is used to construct the central part of the posterior lamella, and will directly overlie the cornea. We used a graft sized approximately 6 mm X 15 mm. Next, the extent of the remaining defect on each side of the central graft is measured and an appropriately sized HPG is harvested. An oral denture is used for one week to aid healing. The full surface of the HPG is thinned to 70% thickness thereby removing the keratinized mucosa, using a diamond burr (Figure 1, top right). This step also helps match the HPG's thickness to that of the tarso-cunjunctival graft. The HPG is divided. placed on either side of the tarsal graft and sutured using 5–0 Polyglactin (Ethicon, Inc., Somerville, NJ) with split thickness passes**.** The composite graft is sutured to the medial and lateral canthal remnants using the same sutures and the stump of the levator aponeurosis is sutured to the superior edge of the composite graft. If the levator aponeurosis remnant is too short to be attached without causing lagophthalmos, an additional HPG can be placed superiorly. When needed, a mucous membrane graft is harvested from the lower lip and sutured in the superior fornix using a 7–0 polyglactin suture.

Next, the Anterior lamella is reconstructed by advancing a myocutaneous flap from the upper eyelid over the composite graft and secured to the reconstructed eyelid margin using 7–0 polyglactin sutures. Antibiotic ointment is instilled and a pressure patch is applied for one week. If a mucous membrane graft was used, a symblepharon ring conformer is inserted. A typical patient is presented in Figure 1 top right and bottom panels.

## Outcomes

This retrospective study was approved by the ethics committee of the University of Miami and the study adhered to the tenets of the Declaration of Helsinki. Data on eight patients who were treated using the described technique by a single surgeon was extracted. All patients had a total upper eyelid defects with a long horizontal component including most of the canthal tendons and sufficient upper eyelid anterior lamella. Patient demographics, indications for surgery, treatment characteristics and surgical outcomes are summarized in Table 1.

**Table 1:** Patient demographics, treatment characteristics and surgical outcomes.

Case	Age	Gender	Side	Diagnosis	Mucous Membrane use?	Cosmetically acceptable, comfortable, Post-op Eyelid Function	Complications	Additional Surgeries	F/U (Months)
**1**	77	F	OS	Seb CCa		Yes	Contracted fornix	Mucous Membrane Graft	54
**2**	79	F	OD	Seb CCa	+	Yes	–		29
**3**	72	F	OS	Seb CCa		Yes	–		84
**4**	75	M	OS	Melanoma	+	Yes	Corneal epithelial defect		21
**5**	78	F	OD	Seb CCa		Yes	–		63
**6**	84	M	OS	Seb CCa	+	Yes	–		8
**7**	80	F	OS	Seb CCa	+	Yes	–		26
**8**	68	F	OD	Seb CCa		Yes	–		106

F- Female; M- Male; F/U- follow up; Seb CCa- sebaceous cell carcinoma; MMG- mucous membrane graft; POM- Post-operative month.

We saw that all grafts did well with minimal shrinkage at a mean follow up of 48 months (range 8–106). Good eyelid-globe apposition, which can be problematic with HPG grafts and lead to corneal irritation,^3^ was achieved in all patients. The reduced thickness of the HPG aided in achieving smooth eyelid appearance. No donor site morbidity was seen. One patient had a forniceal contraction that required a mucous membrane graft, and one patient developed a corneal epithelial defect which resolved with conservative treatment.

Using this novel approach, patients were treated in a single sitting, avoiding temporary visual axis occlusion. The creation of a long, pliable, concave posterior scaffold helped avoiding additional surgical manoeuvres and contributed to creating a tension-free, aesthetically pleasing and functioning eyelid.

The part of the composite graft that is in direct contact with the cornea is the tarso-conjunctival graft which provides a like-for-like substitute, contributing to the low rates of ocular surface complications which can sometimes be seen in these patients.^4^^,^^5^ Aesthetically, reforming a natural contour to the eyelid can be challenging with a tough HPG. This technique emphasizes thinning the HPG and adds two additional bending points along the posterior lamella at the tarsus-HPG junction, which contribute to good eyelid-globe apposition and to a more natural looking eyelid.

Disadvantages include three surgical sites and the need for enough anterior lamella allowing an advancement flap.

In summary, this technique adds to the surgeon's armamentarium when tackling a total upper eyelid defect, regaining eyelid function and offering a good aesthetic outcome.

## Declaration of Competing Interest

None declared.
